# Intracardiac Metastasis From a Testicular Yolk Sac Tumor With Brain and Pulmonary Involvement: A Multimodality Management Case Report

**DOI:** 10.1002/ccr3.73528

**Published:** 2026-09-16

**Authors:** Danial Fazilat‐Panah, Masume Masudian, Mina Heidarian

**Affiliations:** ^1^ Cancer Research Center, Health Research Institute Babol University of Medical Sciences Babol Iran; ^2^ Cancer Research Center Semnan University of Medical Sciences Semnan Iran

**Keywords:** brain metastasis, intracardiac metastasis, multimodality treatment, non‐seminomatous germ cell tumor, pulmonary metastasis, testicular yolk sac tumor

## Abstract

Cardiac metastasis from non‐seminomatous germ cell tumors (NSGCTs) is exceedingly rare and represents an aggressive disease course. We report a 37‐year‐old man with a testicular yolk sac tumor who initially declined adjuvant therapy after orchiectomy. He later developed pulmonary metastases treated with BEP chemotherapy, followed by progressive disease with brain involvement requiring emergent decompressive craniectomy and whole‐brain radiotherapy. Salvage TIP chemotherapy was administered. Persistent pulmonary disease led to thoracotomy, during which a discrete metastatic deposit in the right atrium was identified and surgically resected. Histopathology confirmed metastatic yolk sac tumor in all sites. The patient subsequently received maintenance therapy with oxaliplatin and bevacizumab. Follow‐up has been based on clinical assessment and serial tumor marker monitoring, demonstrating ongoing biochemical and clinical stability for approximately 6 months following initiation of maintenance therapy, with the patient remaining on treatment at the time of this report. This case highlights the unpredictable metastatic behavior of NSGCTs and underscores the importance of multidisciplinary management in achieving disease control in rare and life‐threatening metastatic presentations.

## Key Clinical Message

Cardiac metastasis from testicular yolk sac tumor is exceptionally rare. Multimodality imaging is essential for accurate diagnosis and differentiation from thrombus. Aggressive multidisciplinary management, including surgery, chemotherapy, and radiotherapy, may achieve meaningful disease stabilization even in advanced metastatic presentations, though longer‐term follow‐up is needed to confirm the durability of this response.

## 1 Introduction

Testicular cancer is among the most common solid tumors in young and middle‐aged men. It is generally classified into two main types: seminomatous and non‐seminomatous germ cell tumors (NSGCTs) [1]. The non‐seminomatous group is diverse and tends to have a more aggressive clinical course, often spreading earlier and more widely than seminomas. Most patients diagnosed with NSGCTs are men between 20 and 40 years old. Despite advances in multimodal treatments, metastasis to other organs significantly worsens the prognosis and poses a challenging clinical scenario [2, 3].

The metastatic spread of NSGCTs typically follows a relatively predictable pattern. The lungs are the most common site of metastasis, observed in approximately 50% of patients at diagnosis, followed by the liver, brain, and skeletal system. Brain metastases are less frequent but are well recognized in advanced stages of disease and are associated with poor prognosis [2].

Cardiac metastasis in NSGCTs is exceedingly rare, reported in fewer than 1% of cases, predominantly based on autopsy series and rare case reports. For example, the largest autopsy study suggested that less than 1% of testicular cancer patients have intracardiac metastases. Such metastasis typically involves the right side of the heart but can rarely affect left‐sided cardiac structures [4, 5].

## 2 Case History/Examination

### 2.1 Patient Information and Initial Presentation

A 37‐year‐old man without any past medical history presented in 2021 with testicular pain and swelling. Scrotal ultrasonography revealed a solid intratesticular mass. The patient underwent radical orchiectomy.

Histopathology confirmed a mixed germ cell tumor, composed of approximately 70% post‐pubertal type yolk sac tumor and 30% teratoma. The tumor was confined to the testis, with no lymphovascular invasion or spermatic cord involvement. Preoperative AFP was 200 ng/mL, while β‐hCG and LDH were within normal limits.

Based on the pathological findings (tumor confined to the testis without lymphovascular invasion or spermatic cord involvement), the tumor was classified as pT1. As the patient declined further staging workup and adjuvant therapy, complete nodal and marker‐based staging (S‐stage) could not be determined at that time.

Adjuvant chemotherapy was recommended following orchiectomy, but the patient declined systemic therapy and did not adhere to regular follow‐up visits. The patient's repeated refusal of recommended treatment throughout the disease course was attributed to a combination of financial constraints, limited health literacy/educational background, and fear of chemotherapy‐related toxicity.

### 2.2 Differential Diagnosis, Investigations and Treatment

#### 2.2.1 Pulmonary Metastases and First Chemotherapy

Approximately 12 months later, the patient re‐presented with cough, dyspnea, and episodes of hemoptysis. Contrast‐enhanced computed tomography (CT) of the chest revealed multiple metastatic pulmonary lesions, including an 80 × 57 mm solid mass with central necrosis and surrounding atelectasis in the left upper lobe, an adjacent 36 × 28 mm lesion, and a 42 × 26 mm cavitary lesion in the left lower lobe. Additionally, a 15 × 12 mm solid mass was identified in the mediastinum adjacent to the main pulmonary artery. Abdominal imaging demonstrated no visceral organ involvement. Cardiac evaluation at that time was unremarkable, with no pleural or pericardial effusion.

The patient received four cycles of bleomycin, etoposide, and cisplatin (BEP) chemotherapy. He experienced clinical improvement, complete resolution of hemoptysis, and a reduction in the size of pulmonary lesions.

#### 2.2.2 Disease Progression and Treatment Refusal

During follow‐up in 2024, serial monitoring demonstrated a rising AFP level accompanied by radiologic progression of pulmonary metastases. Despite recommendations, the patient again declined further systemic therapy.

A few months later, he developed paresthesia in the right‐hand fingers. Brain MRI revealed a metastatic lesion in the left temporoparietal lobe. However, the patient, for the third time during the course of his disease, declined to proceed with the recommended medical treatment.

#### 2.2.3 Emergency Neurosurgery and TIP Chemotherapy

**FIGURE 1:**
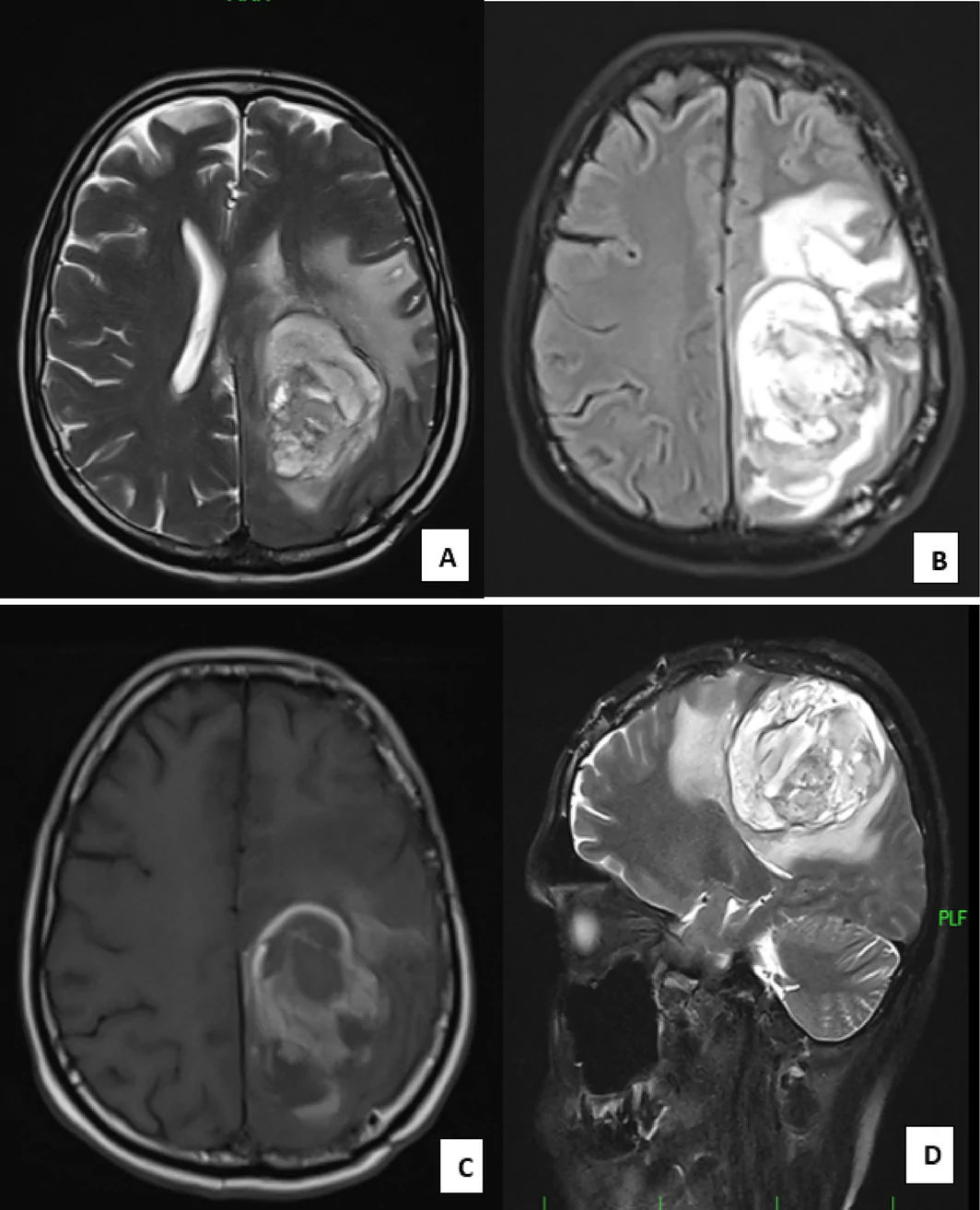
Demonstrating a large ring‐enhancing metastatic lesion in the left parietal lobe with central necrosis.

Approximately 1 year after the AFP rise, the patient presented with a decreased level of consciousness and required urgent neurosurgical intervention. Brain MRI revealed a large left temporoparietal mass with significant perilesional edema and mass effect, causing midline shift (Figure 1).

**FIGURE 2:**
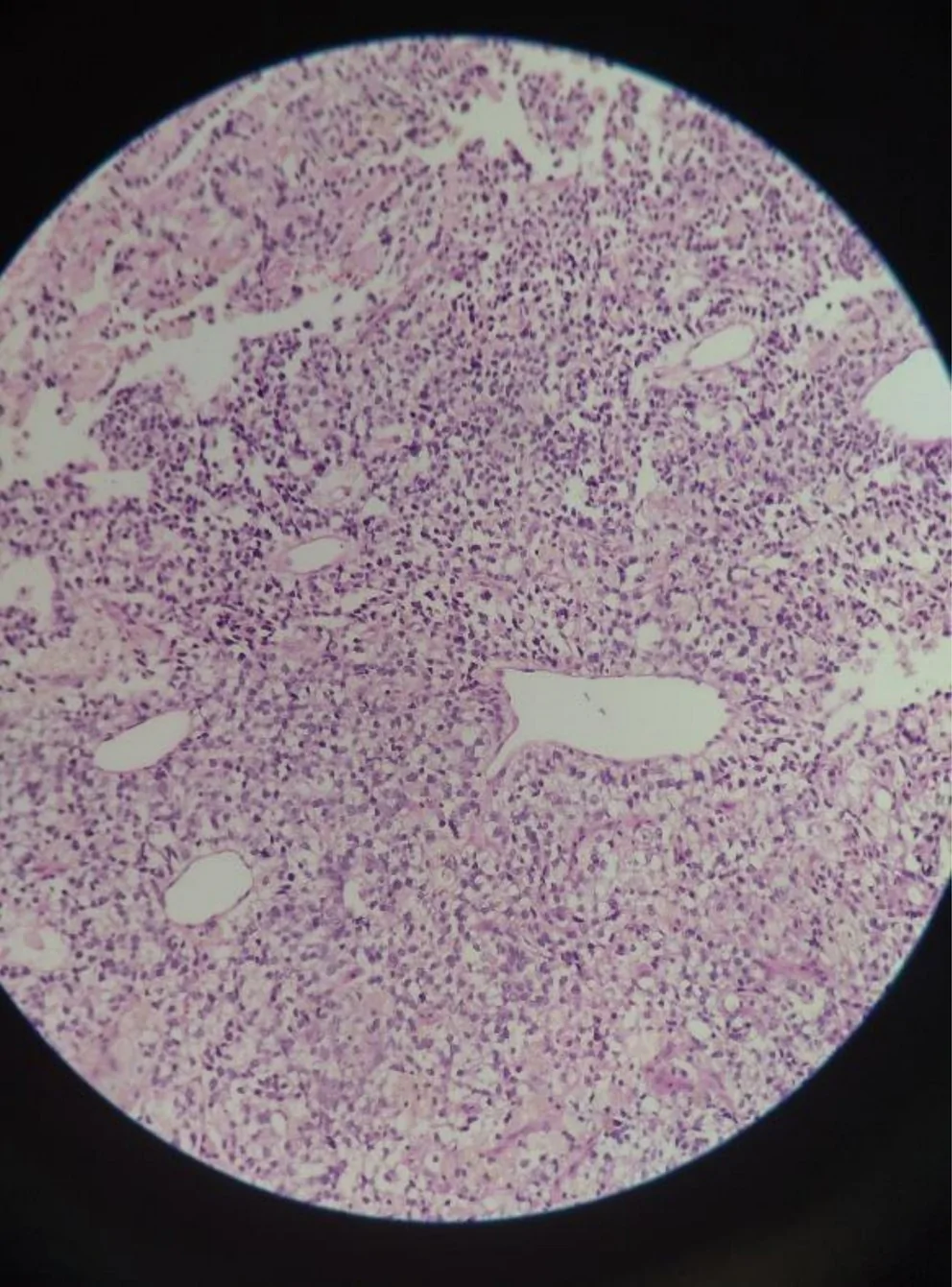
H&E‐stained histology of the brain metastatic lesion confirming yolk sac tumor, showing reticular/microcystic architecture with prominent Schiller‐Duval bodies (arrows) and primitive tumor cells in a loose mesenchymal‐like stroma.

**FIGURE 3:**
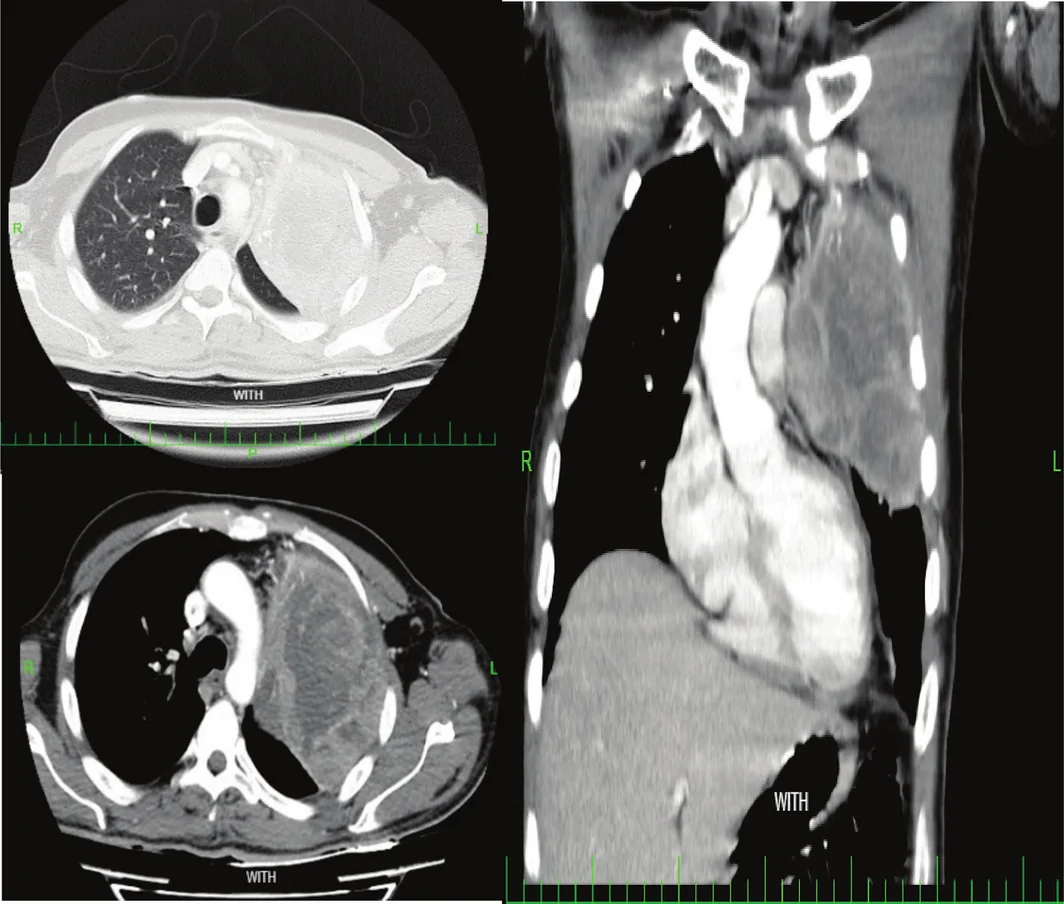
Contrast‐enhanced chest CT.

He underwent decompressive craniectomy. Histopathology confirmed metastatic yolk sac tumor (Figure 2). Following surgery, approximately 1 month after surgery, the patient began four cycles of TIP chemotherapy (Paclitaxel, Ifosfamide, Cisplatin), completed over approximately 2 months (Figure 3).

#### 2.2.4 Residual Pulmonary Lesions and Surgical Resection

**FIGURE 4:**
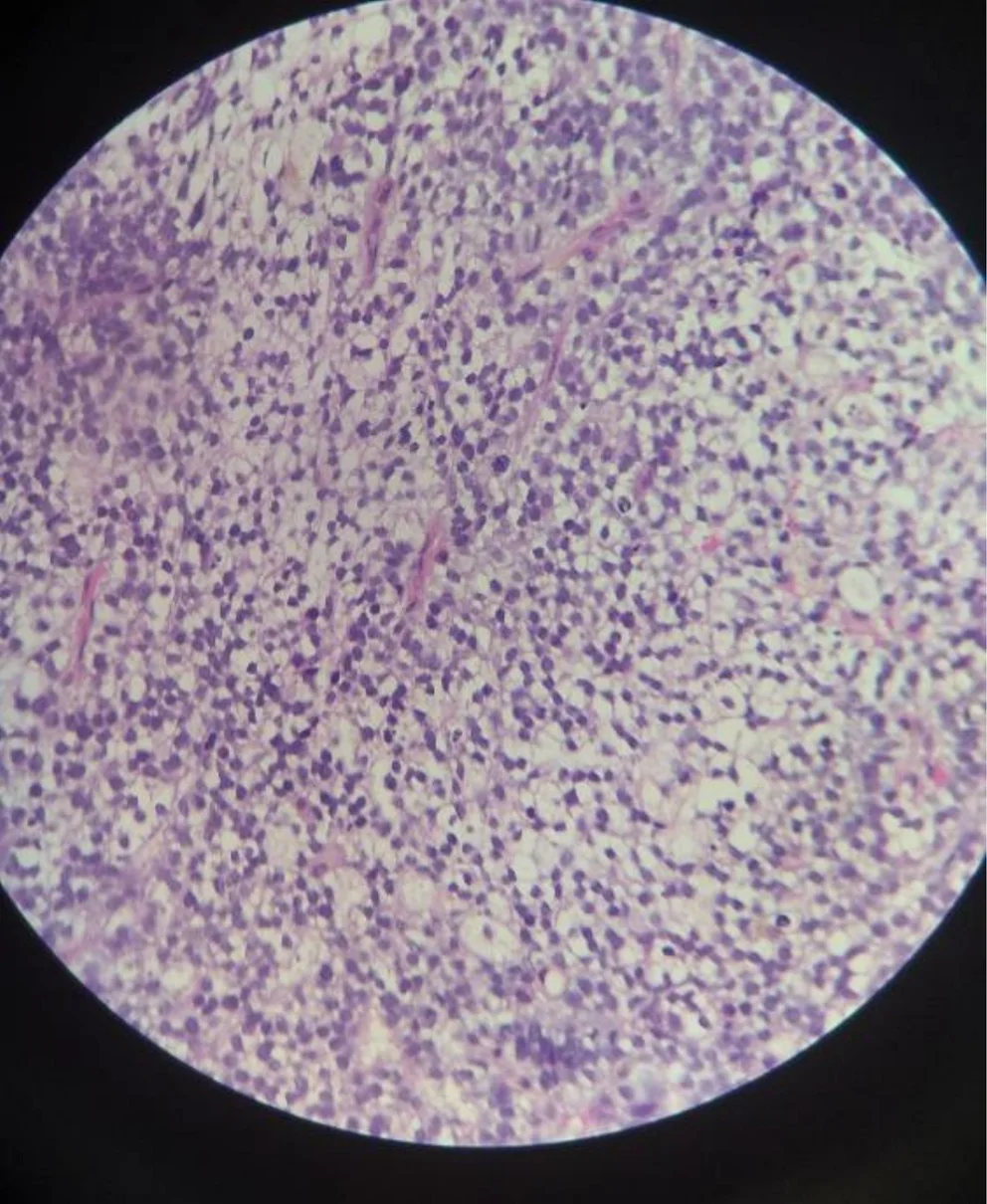
H&E histology of the right atrial metastatic lesion confirming yolk sac tumor, demonstrating reticular/microcystic architecture with primitive tumor cells lining cystic spaces, myxoid stroma, and hyperchromatic nuclei (consistent with the yolk sac component seen in prior pulmonary and cerebral resections).

**FIGURE 5:**
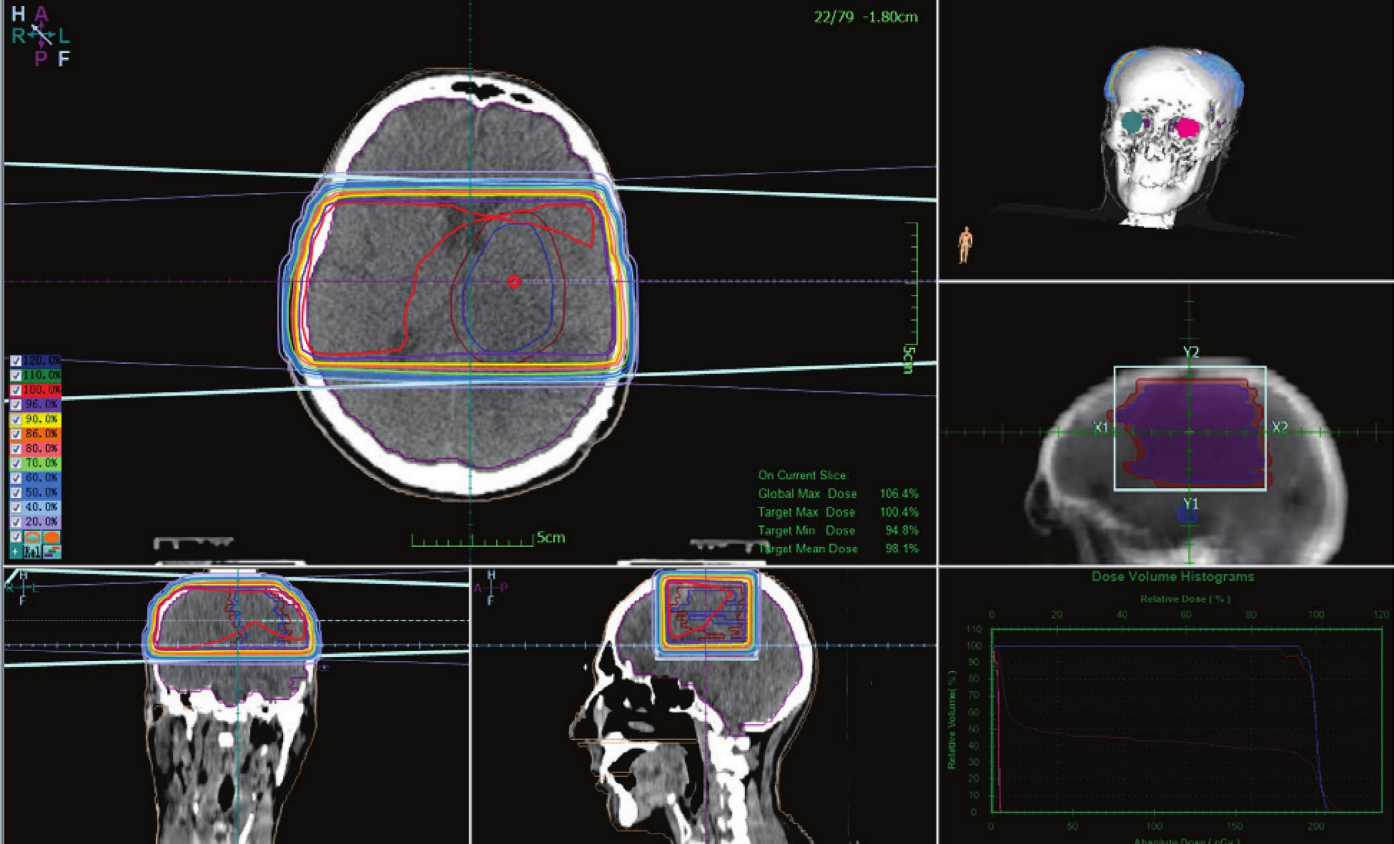
Radiotherapy treatment plan.

Approximately 1 month after completing TIP chemotherapy, follow‐up CT of the chest revealed a persistent dominant pulmonary mass with stable dimensions compared to previous imaging (Figure 3). The patient underwent thoracotomy and surgical resection of the pulmonary mass. A separate, discrete metastatic deposit measuring 50 × 50 × 25 mm was also identified in the right atrium, discontinuous from the primary pulmonary lesion, and was surgically excised. The precise route of hematogenous spread to the right atrium (e.g., via the systemic venous circulation) could not be definitively established based on available operative and pathological documentation. Histopathology confirmed yolk sac tumor involvement of the right atrium (Figure 4). Approximately 1 month after thoracotomy, the patient received whole‐brain radiotherapy at a total dose of 30 Gy (Figure 5).

#### 2.2.5 Maintenance Therapy

Approximately 1 month after completing whole‐brain radiotherapy, the patient was initiated on maintenance therapy with oxaliplatin and bevacizumab, based on a published Phase II clinical trial regimen for refractory germ cell tumors [6].

## 3 Conclusion and Results (Outcome and Follow‐Up)

At the time of this report, the patient has been on maintenance therapy for approximately 6 months. Follow‐up has been based on clinical evaluation and serial tumor marker monitoring. Serum alpha‐fetoprotein levels have remained stable without significant elevation. Clinically, the patient reports no new neurological or cardiopulmonary symptoms, and his general condition has remained stable, with no biochemical evidence of overt disease progression. The sequence of diagnostic, surgical, and systemic treatment events—along with corresponding clinical outcomes—is summarized in Table 1.

**TABLE 1:** Summary timeline of key clinical events, presented relative to the time of orchiectomy.

Time (relative to orchiectomy)	Event
Month 0	Orchiectomy; declined adjuvant therapy
~Month 12	Pulmonary metastases; BEP × 4
~Year 3	AFP rise, radiologic progression; declined therapy
~Year 4	Paresthesia, brain MRI lesion; declined therapy again
Same period	Decreased consciousness; emergent decompressive craniectomy
+1 month	Start TIP ×4 (~2 months duration)
+1 month	Persistent pulmonary lesion; thoracotomy with atrial mass resection
+1 month	Whole‐brain radiotherapy (30 Gy)
+1 month	Initiation of maintenance therapy (oxaliplatin/bevacizumab)
+6 months (ongoing)	Continued clinical/biochemical stability on maintenance therapy

## 4 Discussion

This case highlights the aggressive and unpredictable metastatic behavior of mixed non‐seminomatous germ cell tumors (NSGCTs), especially when dominated by yolk sac tumor histology. Pulmonary and CNS metastases are uncommon at presentation but are recognized in patients with delayed treatment or chemoresistance.

Early multimodal intervention—including timely orchiectomy, platinum‐based chemotherapy, and vigilant follow‐up—is critical for improving survival outcomes [2, 7]. In this case, the patient initially presented with localized disease but declined adjuvant therapy and follow‐up related to financial limitations, low health literacy, and fear of chemotherapy toxicity, which likely permitted early hematogenous dissemination and contributed to chemoresistance.

Pulmonary metastases are the most common site of spread in testicular NSGCT, and initial treatment with BEP chemotherapy led to symptomatic improvement and partial radiologic response. However, the patient subsequently developed neurological symptoms due to a metastatic lesion in the left temporoparietal lobe. Brain metastases in germ cell tumors are uncommon, generally occurring after pulmonary involvement, and are often associated with poor prognosis [8].

Urgent decompressive craniectomy was required to manage life‐threatening intracranial hypertension, consistent with prior reports supporting surgical intervention in selected patients with symptomatic brain metastases [9].

Salvage chemotherapy with TIP (Paclitaxel, Ifosfamide, Cisplatin) combined with whole‐brain radiotherapy stabilized CNS disease, demonstrating the regimen's effectiveness in relapsed or chemo‐resistant NSGCT [10].

Persistent pulmonary lesions and the discovery of a discrete metastatic deposit in the right atrium, anatomically separate from the primary pulmonary lesion, underscore the rarity and hematogenous nature of cardiac involvement in germ cell tumors. Cardiac metastasis from testicular non‐seminomatous germ cell tumors is exceptionally rare but associated with life‐threatening risks such as obstruction or embolism [11]. Literature reports underscore that aggressive surgical resection can be beneficial when feasible, but overall survival remains suboptimal due to the challenges of achieving complete disease control with chemotherapy alone [12, 13]. In this case, thoracotomy and resection of cardiac lesions were achieved, illustrating the feasibility of aggressive surgical intervention in selected patients.

In our case, following craniotomy with resection of the brain metastasis, whole‐brain radiotherapy, and thoracotomy with resection of the right atrial mass, the patient was initiated on maintenance therapy with oxaliplatin and bevacizumab [6]. Remarkably, despite the poor prognosis and extensive metastatic disease, he has remained clinically stable for approximately 6 months following initiation of maintenance therapy (approximately 8 months after thoracotomy), and continues to be followed at the time of this report. Longer‐term follow‐up will be necessary to determine the durability of this response.

This case emphasizes several important clinical lessons. First, clinicians should maintain a high index of suspicion for rare metastatic sites in NSGCT patients presenting with new neurological or cardiopulmonary symptoms. Second, multimodal therapy—including chemotherapy, radiotherapy, and surgery—is critical for disease control in aggressive or atypically metastatic germ cell tumors. Third, salvage regimens such as TIP can provide meaningful responses even in chemo‐resistant cases, and urgent neurosurgical intervention can be lifesaving in patients with brain metastases. Additionally, maintenance therapy with oxaliplatin and bevacizumab may be beneficial in selected patients with chemo‐resistant disease. Finally, patient adherence to recommended therapy and close follow‐up are essential to prevent rapid disease progression and unusual metastatic patterns.

Overall, this case contributes valuable insight into the management of NSGCT with rare, simultaneous metastases to the brain, lungs, and heart, highlighting the need for individualized, multidisciplinary care in complex presentations.

## 5 Limitation

A limitation of this case is the unavailability of complete serial tumor marker data (including post‐orchiectomy AFP kinetics and marker levels at the time of recurrence), which precluded formal IGCCCG risk stratification. This gap itself reflects the patient's repeated loss to follow‐up, which is discussed as a contributing factor to disease progression.

A further limitation of this report is the lack of detailed intraoperative documentation regarding the surgical technique used for resection of the right atrial lesion (e.g., use of cardiopulmonary bypass, extent of atrial wall involvement). We recommend that future reports of similar rare intracardiac metastases follow structured surgical reporting guidelines, such as the PROCESS guidelines, to maximize the educational value of the surgical description for readers managing comparable cases.

## Author Contributions

**Danial Fazilat‐Panah:** conceptualization, data curation, formal analysis, investigation, writing – original draft, writing – review and editing. **Masume Masudian:** conceptualization, data curation, formal analysis, investigation, writing – original draft, writing – review and editing. **Mina Heidarian:** conceptualization, data curation, formal analysis, investigation, writing – original draft, writing – review and editing.

## Funding

The authors have nothing to report.

## Consent

Written informed consent was obtained from the patient for publication of this case report and accompanying clinical information and images. Ethical approval for publication of this case was granted by the Ethics Committee of Babol University of Medical Sciences, Iran. Ethics code: IR.MUBABO.REC.1404.194.

## Conflicts of Interest

The authors declare no conflicts of interest.

## Data Availability

No datasets were generated or analyzed during the current study. All relevant clinical information is included in the manuscript.
